# Social relationship modulates advisor’s brain response to advice-giving outcome evaluation: Evidence from an event-related potential study

**DOI:** 10.3389/fnins.2022.1062095

**Published:** 2022-11-23

**Authors:** Can Zhang, Ruiwen Tao, Hanxuan Zhao, Kexin Zheng, Mengge Dai, Sihua Xu

**Affiliations:** ^1^Center for Magnetic Resonance Imaging Research and Key Laboratory of Applied Brain and Cognitive Sciences, Shanghai International Studies University, Shanghai, China; ^2^College of International Business, Shanghai International Studies University, Shanghai, China; ^3^School of Education, Huaibei Normal University, Huaibei, China; ^4^Anhui Engineering Research Center for Intelligent Computing and Application on Cognitive Behavior, Huaibei Normal University, Huaibei, China

**Keywords:** advice-giving, social relationship, FRN, P3, outcome evaluation

## Abstract

**Introduction:**

Advice-giving is a double-edged sword in social interaction, which could bring benefits or considerable losses for the advisee. However, whether the social relationship affects the time course of advisor’s brain response to outcome evaluation after the advice-giving remains unclear.

**Methods:**

In the present study, we used event-related potentials (ERPs) to investigate the modulation of social relationships on advisor’s outcome feedback processing after the advice-giving and related neural activities.

**Results:**

The results showed larger feedback-related negativity (FRN) to a loss than to a gain both when the friends accepted and rejected the advice, whereas this effect only existed when the strangers rejected the advice, but not when they accepted it. In contrast, the P3 results demonstrated the enhanced neural sensitivity when the strangers accepted the advice than rejected it despite leading to a loss, while a larger P3 amplitude was found when the friends accepted the advice than rejected it and brought a gain. The theta oscillation results in the friend group revealed stronger theta power to loss when the advisee accepted the advice than rejected it. However, this effect was absent in the stranger group.

**Discussion:**

These results suggested that outcome evaluation in advice-giving was not only influenced by feedback valence and social reward, but also modulated by social relationships. Our findings contributed to the understanding of the neural mechanisms of advice-giving outcome evaluation in a social context.

## Introduction

Advice-giving is a widely existing kind of joint decision-making in which an advisor provides suggestions on choice options to the advisee, and the latter is assigned as the decision-maker to choose whether to take the advice that may cause a bad or terrific outcome. In social life, people often give advice to friends, families, and strangers who encounter a dilemma or risky decision-making options. They may react differently and modulate their strategy for offering their predictions in order to maximize their influence when the advisees accept or reject the advice and receive different outcomes (Bayarri and Degroot, 1989). In recent years, research on advice-giving has become an emerging topic, and gradually attracted attention from the field of decision-making (Ache et al., 2020). For example, Belkin and Kong (2018) investigated the effect of advisees’ choices on advice-giving, founding that advice rejection attenuated advisors’ prosocial motivations, and advice acceptance triggered social reward. The enhanced social reward, one of two reward types, involving social affiliation and reputation in the context of social reciprocal interaction, had an important impact on decision-making (Mobbs et al., 2015; Li et al., 2020a).

Researchers have recently explored the effect of social interaction cues, such as social influence and social information, on advisors’ performance in advice-giving. For example, Hertz et al. (2017) found that advisors’ choices were driven by social influence in an advice-giving game. Accordingly, the social influence of decision-makers on advisors leads to the following four conditions: the advice is accepted and decision-makers receive a gain, the advice is accepted but leads to a loss, the advice is rejected and leads to a loss, and the advice is rejected while decision-makers receive a gain (Mobbs et al., 2015; Li et al., 2020a). Explanations were also offered for the four situations, such as the enhanced social reputation for conditions that the advice was accepted and led to a monetary earning, and that the advice was rejected and led to a monetary loss (Li et al., 2020a; Pegg et al., 2022). A recent psychophysiology study using a two-option advice-giving task found larger feedback-related negativity (FRN) amplitudes to error processing when the advice was rejected (Li et al., 2020a), indicating the strong sensitivity to social reputation. However, the P3 effect was absent when advisors obtained social rewards in the late stage (Li et al., 2020a), which was inconsistent with prior studies which found that P3 was a promising neural indicator of reward processing on social acceptance (Ait Oumeziane et al., 2017; Pegg et al., 2022).

These inconsistent results showed the complexity of the effect of social feedback on outcome evaluation in advice-giving, and other plausible explanations were needed. For example, previous studies mainly focused on advice-taking rather than advice-giving. It was found that participants preferred to devaluate bad advice provided by a human advisor rather than a digital agent, reflecting a critical reevaluation process in advice-taking (e.g., Goodyear et al., 2016). However, extant studies mainly focus on the advice utilization from different sources in the role of decision-maker, but how the advisors respond to different advisees in advice-giving and subsequent outcome evaluation remains unclear. Specifically, it is unclear whether advisors’ sensitivities to decisions and outcomes resulting from socially close advisees differ from that resulting from socially distant individuals. In addition, although previous studies have found that social relationship plays a pivotal role in outcome evaluation (Zhang et al., 2019), there was little research on this effect across the advice-giving context. An examination of this issue could enrich our knowledge about determinants of social interaction in interpersonal situations.

Answering these research calls, drawing on the construal level theory (CLT) in the present study could help to fill the above gaps. The CLT theory posits that people often make decisions based on the psychological representation of interpersonal relationship, such as social distance, and they also have different risk propensities when giving advice to strangers (with high-level construal) and making risky decisions for themselves (with low-level construal) (Trope and Liberman, 2003, 2010). Specifically, advisors tend to evaluate risky options with abstract high-level construal in advice-giving, which lead to a self-other discrepancy between themselves and a distant advisee (Danziger et al., 2012; Thorsteinson et al., 2021). Furthermore, if outcomes were presented to advisors and advisees at the same time, advisors would feel responsible for their recommendations, which could reduce the psychological distance between both sides and lower the construal level even if their partners were unfamiliar with advisors (Leiser et al., 2008). The investigation of neural activity on self-other decision-making using electroencephalogram (EEG) recording reported that the individual’s neural activities to other’s gain or loss were affected by social relationships in different stages, labeled by FRN and P3, involving cognitive processing related to reward valence, expectancy violation, attention resource allocation (Leng and Zhou, 2010, 2014; Zhang et al., 2019, 2021). The FRN has been regarded as an important ERP component in decision-making involving outcome evaluation and feedback processing, peaking at 200-300 ms after the feedback delivery (Xu et al., 2020). The FRN acts as a promising indicator of unexpected outcome or negative prediction error in decision-making (Holroyd and Coles, 2002; Xu et al., 2020). P3 peaks at 300-600 ms after the feedback stimuli onset, and has been found to be related with reward processing and motivational function of feedback valance on outcome evaluation in decision-making (Nieuwenhuis et al., 2005; Zhang et al., 2022). In addition, the emerging application of time-frequency analyses reveals a potential for tracking and assessing oscillation activities in the theta band temporally overlapping ERPs in decision-making (Bachman et al., 2022). Several studies found that feedback-related theta-band activity (3-7 Hz), widely localized over mid-frontal regions (Hu et al., 2018), was associated with outcome expectancy and reward magnitude, signaling unexpected negative outcomes compared to expected positive outcomes in the context of monetary and social decision-making (Hajihosseini et al., 2012; Cristofori et al., 2013). However, despite a great deal of research on social relationships and self-other decision-making (Li et al., 2020b; Wang et al., 2021), there has been little examination of the effect of social relationship on social feedback processing and outcome evaluation in the perspective of advisor.

Taken together, previous studies have demonstrated that advisors reacted differently to advisees’ choices and decision-making outcomes in social interaction, but the modulation of social relationship cues on advice-giving outcome evaluation was unknown. To fill this gap, the current study focused on advisors’ brain response to outcome evaluation involving social reward using a modified card-guessing task, and examined the modulation of social relationships. The two-option card-guessing task was used to simulate the advice-giving game. Because of advantages in tracking phasic brain activities with high temporal resolution and low cost (Hu and Shealy, 2020; Fu et al., 2022), the event-related potentials (ERPs) and the time-frequency approach were adequate to collect and analyze the neural dynamics of decision-making in social context, which could acquire a deep and precise measurement of subject’s implicit cognitive fluctuations (Wang C. et al., 2020). Therefore, we used ERPs and time-frequency analyses to unravel the neural dynamics of advice-giving outcome evaluation, which were signaled by FRN, P3, and theta power (Hajihosseini et al., 2012; Hu et al., 2017; Wang et al., 2017; Li et al., 2020a). Based on previous findings, we predicted that the FRN, P3 amplitudes, and theta power are modulated by the social relationships between advisors and their partners. The following hypothesis is proposed:

**H1**: There is larger FRN amplitudes in response to a loss, and the variation in the P3 amplitudes following advice acceptance are larger than those following the advice rejection.

Given that FRN, P3 responses and theta activities were elicited differently following a gain and a loss resulting from a stranger or a friend (Leng and Zhou, 2010; Hu et al., 2018; Zhang et al., 2019). We further propose hypothesis 2:

**H2**: The FRN and P3 effects on gain and loss reveal a reversed pattern between the two relationships, and the theta oscillation to other’s gain and loss also reveals a friend-stranger difference.

## Materials and methods

### Participants

A total of 60 participants (*n* = 36 females, mean age = 21.55 ± 2.86) were recruited for the present study. The EEG data of six participants were excluded since over 50% of EEG trials were eliminated due to technical problems. Thus, the data of 54 participants (*n* = 32 females, mean age = 21.65 ± 2.50) were used in the current study, including 27 in the friend group and 27 in the stranger group. Participants in the friend group reported how long they had been familiar with their partners to identify whether they were in a firm friendship before the formal experiment. The others in the stranger group also answered the question to confirm they were unacquainted with each other. Participants in the friend group had been in a solid friendship, and they had been familiar with each other approximately for half a year (*M* = 5.47, *SE* = 1.43). The latter in the stranger group confirmed that they were absolutely unfamiliar. In addition, subjects in both groups were also asked to finish the Inclusion of the Other in the Self scale (Aron et al., 1992; Feng et al., 2020) to report the extent to how the subjects feel the connection with their partners as a manipulation check of social relationship. The independent-sample *t*-test showed that participants in the friend group rated higher level of self-other inclusion (*M* = 4.29, *SE* = 1.67) than that in the stranger group (*M* = 1.00, *SE* = 0.00), *t*(52) = 19.71, *p* < 0.001. The results demonstrated the manipulation of social relationship was effective.

All subjects had normal or corrected vision, were right-handed, and without a history of neurological or mental disorders. This research was approved by the scientific review committee of the Laboratory of Applied Brain and Cognitive Sciences at Shanghai International Studies University. All participants gave their written, informed consent, and were informed of their rights to withdraw from participation before the formal experimental procedure, in accordance with the 1964 Declaration of Helsinki. Before the formal experiment, participants were told that they could obtain 60 Chinese yuan (about $10.00) for their participation and an additional earning of up to 30 yuan (about $5.00) based on their performance.

### Experimental task and procedure

In the current study, we utilized a modified version of the card-guessing task in which an advisor provided a suggestion to the advisee (Li et al., 2020a). We used a 2 × 2 × 2 mixed design to investigate the modulation of social relationship on advice-giving outcome evaluation, in which the social relationship with the advisee (friend vs. stranger) served as a between-subjects factor, and the advisee’s decision (accept the advice vs. reject the advice) and decision-making outcome (gain vs. loss) served as within-subject factors. According to the CLT, we manipulated the social relationship between the advisor and the advisee, and to explore whether the subjects tend to evaluate outcomes and social feedbacks with concrete low-level construal or abstract high-level construal, which could reveal a choice preference in decision-making. Participants were randomly assigned to either the friend group or the stranger group. Specifically, participants in the friend group were told to bring a mutual familiar friend of the same gender and in a consolidated friendship, forming dyad members. Otherwise, they were not allowed to attend the experiment. All dyad members reported how long they had become familiar friends, and finished a check to rate self-other overlap with their friends, respectively. On the other hand, every participant in the stranger group was randomly matched with an unfamiliar stranger of the same gender as his confederate, and both of the dyads confirmed that they were absolutely unknown with each other.

Before the participation, subjects were asked to wash and blow-dry their hair carefully to ensure the acceptable electrode impedances, and worn the EEG caps after sitting before the screen under the experimenter’s help according to the scalp distribution. After informing the participants of the procedure, the experimenter injected EEG gel into every required electrode on the EEG cap so that the neuroelectrophysiology signals could be easily transferred and measured by the acquisition system (Fu et al., 2022). Before the formal experiment, the subjects were informed of a cover story claiming that they would be assigned to two roles by drawing lots: the advisor and the advisee. The task for the advisor was to select one from two cards presented on the screen as advice provided to the advisee, and his confederate decided to accept or reject this advice. They were told that one of two cards could increase a 10-yuan earning (+10), while the other card would result in a 10-yuan loss (−10). After making a decision, both of them would receive the outcome presented on the screen in every trial at the same time. However, in fact, both of every dyad were assigned to be the advisor in two separate rooms, and two outcomes of casting lots were both “Advisor,” but they were unknown to this manipulation. Therefore, there were four outcomes for every participant they would observe: the advisee accepted his advice and received a gain (+10), the advisee rejected his advice and received a loss (−10), the advisee accepted his advice and received a loss (−10), and the advisee rejected his advice and received a gain (+10). The first two outcomes meant that the advisor offered the right suggestion, and the latter two indicated that the advisor provided wrong advices in the present trial. Participants’ task was to help the advisors obtain more monetary gains by providing suggestions in the risky decision-making.

**FIGURE 1:**
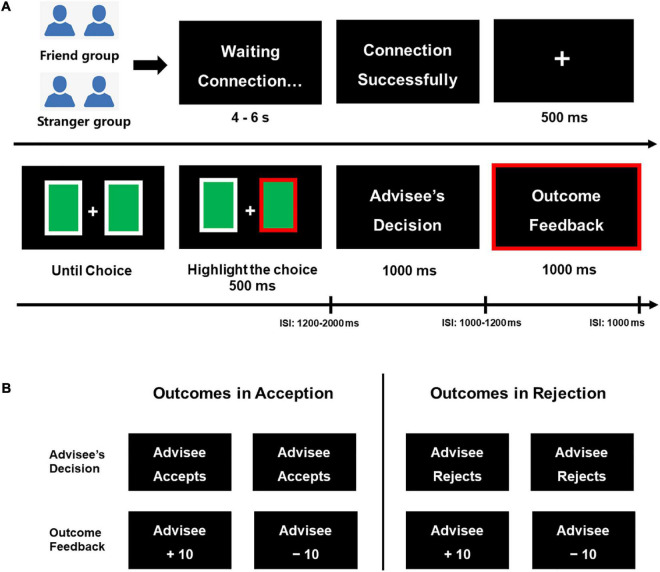
Experimental procedure for the card-guessing task **(A)** and the advisee’s decision, outcome feedback participant could receive in each condition by choosing a card **(B)**.

At the beginning of the experiment, the participants confirmed that they were chosen to be the advisor by drawing lots. They were instructed that they were partaking in an online card-guessing task with his advisee in another room, and two computers were connected in real time. They were presented with a processing interface, and were told that they had to wait 4–6 s for a successful connection. But the interface and subsequent procedures were actually pre-programmed. During the phase of choosing cards, two green cards were firstly presented on the screen, and participants had to choose from the two cards as the advice by pressing the “F” button (representing the left card) or the “J” button (the right card). The chosen card was then highlighted by thick red lines lasting for 500 ms, and participants were told that this advice was sent to the advisee by sharing the present screen simultaneously. In the phase of decision-making, participants observed the fictitious advisee’s choice: accepting the suggestion or rejecting it, which would be displayed on the two screens. In the next phase of displaying outcome feedback, whether the advisee’s choice resulted in a gain (“Advisee +10”) or a loss (“Advisee −10”) would be presented for 1,000 ms, indicating that the advisees would acquire a 10-yuan bonus or suffer 10-yuan loss, while the advisors had no chance to alter these outcomes. The probability of gain and loss was also in a random order which was unknown to participants. Participants in each group were required to complete 10 practice trials to become familiar with the task, and then they had to finish 60 rounds of the card-guessing in each condition which formed 240 trials in total and lasted around 40 min, respectively. We divided the 240 trials into four blocks, retaining 60 trials in each block. Participants had 1 min to rest in the inter-block interval, and they were also informed that they had no time limit for choosing a card from the two options in each trial. The procedure was programmed with E-prime 2.0 (Psychology Software Tools, Inc., Pittsburgh, PA, see Figure 1). After finishing the card-guessing task, all the participants were asked to rate their belief in the cover story and experimental settings. Their answers showed that the manipulation was credible and effective. In the end, participants were informed that the advice-giving interaction was pre-programmed, and their partners’ responses were randomly preset.

### Electroencephalography recording and analyses

The study recorded the electroencephalography (EEG) data from 32 scalp sites using the Brain Product (Gilching, Germany) according to the 10–20 international system. All scalp electrode impedances were less than 10 kΩ, with a sampling rate set at 500 Hz. The FCz served as the online reference electrode. We preprocessed the offline data using Toolbox EEGLAB 12.0.2 (Delorme and Makeig, 2004) and in-house MATLAB scripts. First, the EEG data were re-referenced to the average of the two mastoids and electrode FCz was reinstated. Data were then filtered at a low-pass 30 Hz and a high-pass 0.1 Hz. EEG epochs were segmented from −1,000 to +2,000 ms around outcome feedback (“Advisor +10” and “Advisor −10”) onsets. We used an independent component analysis (ICA) to reject artifacts caused by eyeblinks (de Bruijn et al., 2020). Technical and other noises were also eliminated if the epochs with EEG amplitudes exceeded ± 100 μV before averaging.

In the present study, we analyzed two ERP components extracted from preprocessed data, namely the FRN and the P3. For the FRN, we measured the mean amplitudes at the Fz electrode between 240 and 360 ms following the outcome feedback onset (Hu et al., 2017; Li et al., 2020a; Zhang et al., 2021). For the P3, we measured the mean amplitudes between 340 and 440 ms following the outcome feedback onset (Zhang et al., 2022) at the Pz electrode, which was the most positive parietal site according to previous studies (Yeung and Sanfey, 2004; Pornpattananangkul et al., 2017). The time-frequency analysis was conducted with Letswave7.^1^ For EEG epochs from each single trial, we used continuous wavelets transform (CWT) for time-frequency localization, which could estimate the window width as a function of the measured frequency (Pfurtscheller and Lopes da Silva, 1999). The CWT procedure provide a Morlet-based function that is defined as the product of a complex sine wave and a Gaussian window: ψ(*t*) = *e*^–*t*^2/2^*σ*^2^^ *e*^*j*^2*πωt*^^ (Cohen, 2019), where *t* is time, *ω* is the center frequency, and *σ* is the spread of the Morlet function in time and frequency. Each epoch was baseline corrected (−500 to −200 ms around the feedback onset). Subsequently, theta power activity was averaged for each participant and normalized in the range of 4–7 Hz (Roach and Mathalon, 2008). We extracted the theta from 200 to 500 ms following feedback at the Fz electrode site, which was analyzed in the time window of the FRN. Table 1 shows the number of artifact-free trials that entered analyses in each condition. A 2 × 2 × 2 repeated-measures ANOVAs were performed with outcome feedback (gain vs. loss) and the advisee’s decision (acceptance vs. rejection) as within-subject factors, and social relationship with the advisee (friend vs. stranger) as the between-subjects factor in SPSS 21.0 (SPSS Inc., Chicago, IL). The significance level was set at 0.05. The Bonferroni correction was applied for *post hoc* comparisons.

**TABLE 1:** The number of accepted trials in response to outcome feedbacks following the advisee’s decisions in the friend and stranger groups (*M* ± *SD*).

	Friend group		Stranger group	
	Gain	Loss	Gain	Loss
Acceptance	55.96 ± 4.27	55.85 ± 3.69	55.56 ± 4.82	54.33 ± 5.83
Rejection	55.78 ± 4.29	55.89 ± 3.95	55.48 ± 4.26	55.00 ± 5.31

## Results

In the card-guessing task, the participants as advisors provided suggestions on risky decision-making, but they were passively processed their partners’ choice behaviors and outcome feedbacks. Therefore, we analyzed and reported ERP results in the following sections.

### The feedback-related negativity results

**FIGURE 2:**
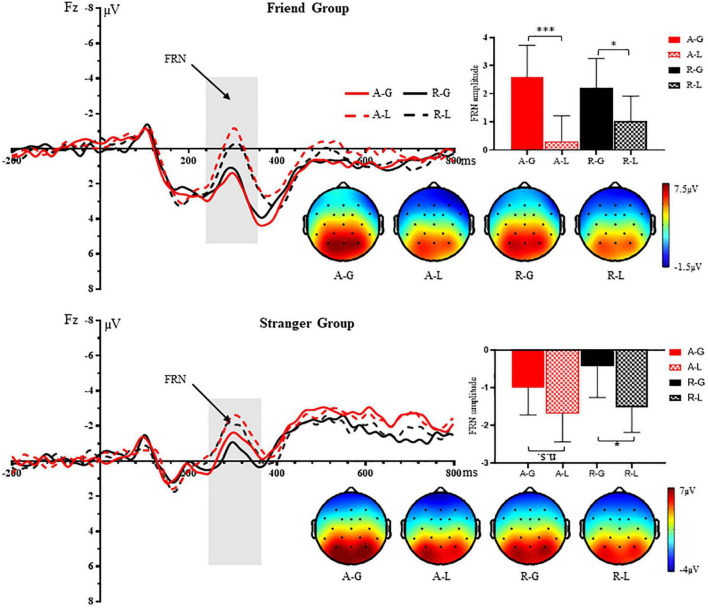
Grand-averaged waveforms, amplitudes and topography from 240 to 360 ms at the Fz electrode in response to four combinations of outcome feedbacks following the advisee’s decisions: acceptance and receiving a gain (A-G), acceptance and receiving a loss (A-L), rejection and receiving a gain (R-G), and rejection and receiving a loss (R-L) in the friend group and the stranger group, respectively. *p < 0.05, ^***^p < 0.001. n.s. = non-significant.

Figure 2 shows the grand average FRN at the Fz electrode in response to positive and negative outcome feedbacks: gain and loss. The three-way repeated-measures ANOVA revealed a significant main effect of outcome feedback, *F*(1, 52) = 16.10, *p* < 0.001, $\eta_{p}^{2}=0.236$, with larger FRN amplitudes elicited by a loss (*M* = −0.46 μ*V*, *SE* = 0.54) than a gain (*M* = 0.85 μ*V*, *SE* = 0.65). In addition, we observed a significant main effect of social relationship with the advisee, *F*(1, 52) = 5.49, *p* = 0.023, $\eta_{p}^{2}=0.096$, indicating that decisions made by a stranger (*M* = −1.16 μ*V*, *SE* = 0.81) elicited larger FRN amplitudes when comparing with a familiar friend (*M* = 1.54 μ*V*, *SE* = 0.81). We also found a significant three-way interaction among outcome feedback, the advisee’s decision and social relationship with the advisee, *F*(1, 52) = 4.40, *p* = 0.041, $\eta_{p}^{2}=0.078.$ The follow-up contrasts showed that in the friend condition, whether the friend accepted or rejected the advisors’ suggestions, FRN amplitudes elicited by a loss were larger than a gain [acceptance: *F*(1, 52) = 17.34, *p* < 0.001, $\eta_{p}^{2}=0.250$; rejection: *F*(1, 52) = 5.53, *p* = 0.023, $\eta_{p}^{2}=0.096$]; while in the stranger condition, decision-makers’ loss elicited larger amplitudes than gain only when the unfamiliar advisees rejected advisors’ suggestions [acceptance: *F*(1, 52) = 1.60, *p* = 0.212, $\eta_{p}^{2}=0.030$; rejection: *F*(1, 52) = 4.67, *p* = 0.035, $\eta_{p}^{2}=0.082$]. These results indicated a stronger sensitivity to negative outcomes whether the friends accepted or rejected the advisors’ suggestions, whereas this effect only existed when their advices were rejected by their unfamiliar confederates and therefore received negative outcomes. We did not observe any other significant main effects or interactions (all *p* > 0.05).

### The P3 results

**FIGURE 3:**
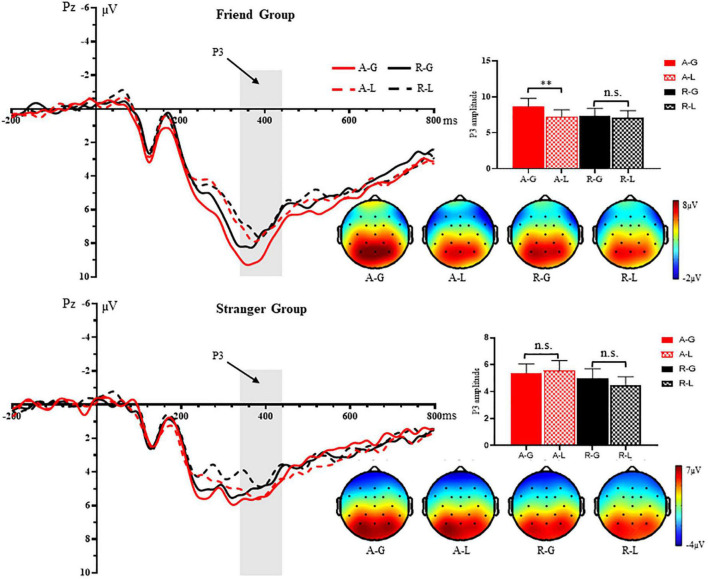
Grand-averaged waveforms, amplitudes and topography from 340 to 440 ms at the Pz electrode in response to four combinations of outcome feedbacks following the advisee’s decisions: acceptance and receiving a gain (A-G), acceptance and receiving a loss (A-L), rejection and receiving a gain (R-G), and rejection and receiving a loss (R-L) in the friend group and the stranger group, respectively. ^**^p < 0.01, n.s. = non-significant.

Figure 3 presents the grand average P3 at the Pz electrode in response to positive and negative outcome feedbacks. The three-way repeated-measures ANOVA yielded a significant main effect of outcome feedback, *F*(1, 52) = 4.14, *p* = 0.047, $\eta_{p}^{2}=0.074$, with larger P3 amplitudes elicited by a gain (*M* = 6.58 μ*V*, *SE* = 0.64) than a loss (*M* = 6.07 μ*V*, *SE* = 0.58); and a significant main effect of the advisee’s decision, *F*(1, 52) = 13.67, *p* = 0.001, $\eta_{p}^{2}=0.208$, with larger P3 amplitudes elicited by acceptance (*M* = 6.68 μ*V*, *SE* = 0.63) than rejection (*M* = 5.97 μ*V*, *SE* = 0.59). In addition, there was a significant main effect of social relationship with the advisee, *F*(1, 52) = 4.23, *p* = 0.045, $\eta_{p}^{2}=0.074$, suggesting that acquainted friendship elicited larger P3 amplitudes than unfamiliar relationship. Importantly, we found a significant three-way interaction among outcome feedback, the advisee’s decision and social relationship with the advisee, *F*(1, 52) = 4.60, *p* = 0.037, $\eta_{p}^{2}=0.081.$ The follow-up contrasts showed that in the friend condition, receiving gain feedback elicited larger P3 amplitudes than loss when participants’ advices were accepted by friends, *F*(1, 52) = 9.16, *p* = 0.004, $\eta_{p}^{2}=0.150$, while the between-feedback difference was non-significant when their advices were rejected. However, in the stranger condition, the difference between gain and loss feedbacks on P3 was non-significant whether participants’ advisees decided to accept or reject their advices. Moreover, we also found a friend-stranger difference in outcome evaluation by the results of outcome feedback × advisee’s decision. Specifically, in the stranger condition, acceptance elicited larger P3 amplitudes than rejection only when they received a loss, *F*(1, 52) = 5.53, *p* = 0.023, $\eta_{p}^{2}=0.096$, and this effect was absent when they received a gain, *F*(1, 52) = 1.47, *p* = 0.231, $\eta_{p}^{2}=0.028$; while in the friend condition, acceptance elicited larger P3 amplitudes than rejection only when they observed a gain, *F*(1, 52) = 13.25, *p* = 0.001, $\eta_{p}^{2}=0.203$, and this effect was absent following a loss, *F*(1, 52) = 0.04, *p* = 0.834, $\eta_{p}^{2}=0.001$. These results suggested that participants’ brain sensitivities to outcome feedback interacted with advisee’s decision, and this effect was modulated by social relationship with advisees. We did not observe any other significant main effects or interactions (all *p* > 0.05) (Table 2).

**TABLE 2:** Summary of the repeated-measures ANOVA performed on FRN, P3, and Theta.

Effect	*Df*	*F*-value	*P-value*	$\eta_{p}^{2}$
**FRN**				
Outcome feedback	(1, 52)	16.10***	<0.001	0.236
Social relationship	(1, 52)	5.49*	0.023	0.096
Advisee’s decision	(1, 52)	1.05	0.311	0.020
Outcome feedback × social relationship	(1, 52)	1.66	0.204	0.031
Outcome feedback × advisee’s decision	(1, 52)	0.95	0.333	0.018
Advisee’s decision × social relationship	(1, 52)	0.11	0.739	0.002
Outcome feedback × Social relationship × advisee’s decision	(1, 52)	4.40*	0.041	0.078
**P3**				
Outcome feedback	(1, 52)	4.14*	0.047	0.074
Social relationship	(1, 52)	4.23*	0.045	0.075
Advisee’s decision	(1, 52)	13.67***	<0.001	0.208
Outcome feedback × social relationship	(1, 52)	1.91	0.173	0.035
Outcome feedback × advisee’s decision	(1, 52)	0.37	0.547	0.007
Advisee’s decision × social relationship	(1, 52)	0.03	0.861	0.001
Outcome feedback × social relationship × advisee’s decision	(1, 52)	4.60*	0.037	0.081
**Theta**				
Outcome feedback	(1, 52)	4.60*	0.037	0.081
Social relationship	(1, 52)	4.39*	0.041	0.078
Advisee’s decision	(1, 52)	0.26	0.615	0.005
Outcome feedback × social relationship	(1, 52)	4.60*	0.037	0.081
Outcome feedback × advisee’s decision	(1, 52)	4.32*	0.043	0.077
Advisee’s decision × social relationship	(1, 52)	0.08	0.781	0.001
Outcome feedback × social relationship × advisee’s decision	(1, 52)	4.91*	0.031	0.086

*p < 0.05, ***p < 0.001.

### Time-frequency results

**FIGURE 4:**
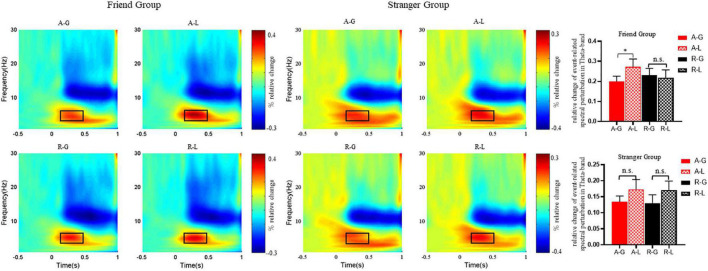
Grand-averaged time-frequency plots at electrode Fz in response to four combinations of outcome feedbacks following the advisee’s decisions: acceptance and receiving a gain (A-G), acceptance and receiving a loss (A-L), rejection and receiving a gain (R-G), and rejection and receiving a loss (R-L) in the friend group and the stranger group, respectively. Black squares indicate time-frequency windows for significant clusters found in the observed data (theta: 0.2–0.5 s at 4–7 Hz). *p < 0.05, n.s. = non-significant.

Figure 4 illustrates the grand-averaged time-frequency plots of theta recorded at Fz. The three-way repeated-measures ANOVA revealed a significant main effect of outcome feedback, *F*(1, 52) = 4.60, *p* = 0.037, $\eta_{p}^{2}=0.081$, with stronger theta power elicited by a loss (*M* = 0.17 μ*V*, *SE* = 0.02) than a gain (*M* = 0.21 μ*V*, *SE* = 0.16), and a significant main effect of social relationship with the advisee, *F*(1, 52) = 4.39, *p* = 0.041, $\eta_{p}^{2}=0.078$, indicating that decisions made by a familiar friend (*M* = 0.23 μ*V*, *SE* = 0.03) elicited stronger theta band activities than a stranger (*M* = 0.15 μ*V*, *SE* = 0.03). More importantly, we observed a significant three-way interaction effect among outcome feedback, the advisee’s decision and social relationship with the advisee, *F*(1, 52) = 4.91, *p* = 0.031, $\eta_{p}^{2}=0.086.$ The follow-up contrasts showed that in the friend group, theta power in the acceptance condition was stronger than that in the rejection condition when they received a loss [*F*(1, 52) = 4.28, *p* = 0.044, $\eta_{p}^{2}=0.076$], while this effect was absent following a gain [*F*(1, 52) = 2.19, *p* = 0.145, $\eta_{p}^{2}=0.040$]. However, in the stranger group, the difference between gain and loss feedbacks on theta power was non-significant whether participants’ advisees accepted or rejected their suggestions. In addition, in the friend group, theta power following a loss was stronger than a gain in the acceptance condition [*F*(1, 52) = 6.71, *p* = 0.012, $\eta_{p}^{2}=0.114$], and this effect was absent in the rejection condition [*F*(1, 52) = 0.215, *p* = 0.645, $\eta_{p}^{2}=0.004$] as well as in the stranger group [acceptance: *F*(1, 52) = 1.81, *p* = 0.185, $\eta_{p}^{2}=0.034$; rejection: *F*(1, 52) = 2.51, *p* = 0.119, $\eta_{p}^{2}=0.046$]. We did not observe any other significant main effects or interactions (all *p* > 0.05).

## Discussion

The present study aimed to examine the modulation of social relationships on advisors’ social feedback processing and outcome evaluation in advice-giving using a modified card-guessing task while EEG was recorded. We also used the time-frequency analysis to assess theta oscillation associated with the time course of advisors’ outcome evaluation. To the best of our knowledge, this is the first study exploring and reporting these relationships. The results showed different neural sensitivities to social feedbacks and outcome evaluation involving socially close and distant relationships, including FRN, P3, and theta power. In the following sections, we discussed the modulation of social relationships and the time course of neural activities related to advisors’ social feedback processing and outcome evaluation.

The FRN results showed more negative FRN after a friend received a loss than a gain both in rejection and acceptance conditions, supporting the proposition in previous work which found that the FRN acted as a negative reinforcement signal when subjects received a failure in decision-making (Holroyd and Coles, 2002; Xu et al., 2020). However, although we observed more negative FRN after receiving a loss than a gain when the stranger rejected the advisor’s advice, this effect was absent in the “stranger-advice acceptance” condition, suggesting that unfamiliar relationship enhanced neural sensitivity to outcomes resulting from social rejection. These results were consistent with previous research on self-other decision-making founding that social rejection cues triggered the FRN amplitudes (Hewig et al., 2008; Pegg et al., 2021). Being rejected by agents with distant social relationships, advisors tended to process information with abstract high-level construal and the violation of reward expectation was larger than the acceptance condition, thus elicited larger variations of FRN amplitude. In addition, lower reward expectation for rejections by strangers would lead to the highest reward positivity in the rejection-gain condition, which is also consistent with proposition that the FRN reflect a reward-related positivity and is absent elicited by non-reward (Proudfit, 2015). On the contrary, accepted by advisees, subjects might be satisfied with relation reputation from strangers, and then reduced the outcome expectation. Therefore, the FRN effects were elicited equally by gain and loss outcomes if the strangers accepted advisors’ suggestions, showing that subjects were likely to pay more attention to social cues including social relationships and social feedback (rejection or acceptance) from distant agents rather than gain-loss outcome in the early stage, which further extended previous ERP studies on advice-giving (Hewig et al., 2008; Li et al., 2020a).

Regarding the P3 results, we found that receiving a gain, as a positive performance feedback, elicited larger P3 amplitudes than loss in the “friend—advice acceptance” condition, reflecting the processing of reward magnitude in the late stage of outcome evaluation. P3 amplitudes were considered to capture an enhanced level of motivational resources and reward valence (Carrillo-de-la-Peña and Cadaveira, 2000; Xu et al., 2020), and previous studies also found that larger P3 amplitudes were associated with the influence of social reward (Li et al., 2020a; Wang D. et al., 2020). It was thought as a social reward for advisors that was accepted by the friend and led to winnings in advice-giving interaction, evoking increased attentional resources and motivational significance toward positive feedback, which induced larger P3 amplitudes. The results provided support for prior studies finding that the P3 was a promising neural indicator of reward processing on social acceptance (Ait Oumeziane et al., 2017; Pegg et al., 2022). However, unlike prior findings on others’ outcome evaluation (Zhang et al., 2019), we found no significant P3 difference between receiving a gain and a loss if the advisors’ agents were strangers. Based on CLT, the possible explanation was that with distant interpersonal relationship, advisors tended to evaluate outcomes with abstract construal level, and reduced the sensitivity to the binary valence of outcomes. This proposition was also supported by our recent evidence on self-other dynamic decision-making finding that the P3 was more insensitive to outcomes when subjects made choices for strangers (Xu, 2021). Inconsistent with our study, the presentation of positive and negative outcomes in Zhang et al. (2019) was simply indexed by “+” and “−,” rather than “+10” and “−10.” This more abstract presentation of the outcome feedback might be the main reason why they failed to observe the influence of interpersonal relationships on P3 amplitudes during the late stage.

Moreover, we also examined the P3 difference between advice acceptance and rejection conditions, and observed a more positive P3 response to a loss in the advice acceptance condition than rejection condition in the stranger group. However, this effect only existed in response to a gain when the advisor’s partner was a close friend, which revealed a relationship discrepancy in outcome feedback processing. People might feel responsible for their advices in acceptance condition, and then became guilty or even regretful when they observed others’ disadvantageous outcomes. Therefore, the decreased social reward threatened subjects’ self-evaluation, which led to high-level affective processing in the late stage (Yeung and Sanfey, 2004; Liu et al., 2021). These results indicated that P3 amplitudes were not only modulated by social relationships involving strangers and friends, but also affected by interpersonal social reward. Our findings also reflected different roles of the FRN and P3 in the early and late stages of feedback processing: the FRN indicating processing related with social feedbacks from different relationships, and the P3 signaling the difference of reward processing with high- and low- level construal, which extended previous findings on self-other decision-making and advice-giving (Leng and Zhou, 2010, 2014; Li et al., 2020a; Liu et al., 2020).

The results of time-frequency analyses showed that theta power played a role in outcome evaluation in decision-making, which was in line with previous studies finding that stronger theta band power was associated with unexpected negative outcomes compared to expected positive outcomes (Hajihosseini et al., 2012; Luft et al., 2013; Zheng et al., 2020). This pattern was also closely associated with variations of the FRN component in feedback processing, and further supported the proposition that the FRN shared a common signature with spectral fluctuation within the theta band (Cavanagh and Shackman, 2015). However, our results on the FRN and theta power were somewhat discrepant. It may be explained by the two dissociable cognitive processes indexed by the FRN and theta power. Specifically, the FRN acts as a dopamine reinforcement learning signal and is sensitive to the reward valence while the activities in the theta band, as a general “alarm,” indexes the need for increased cognitive control (Hajihosseini and Holroyd, 2013; Cavanagh and Shackman, 2015; Rawls et al., 2020). In addition, as predicted, we observed a modulation of social relationships between the advisor and the partner. Previous research has found differential sensitivity of theta power to acceptance and rejection in social interaction contexts, and enhanced theta power was related to social pain (Cristofori et al., 2013; Moore et al., 2021). The current study extended this finding in an advice-giving context, and the friend-stranger difference in theta power may indicate that subjects experienced higher social pain when their suggestions were accepted by their friends but led to a loss, and a distant relationship smoothed this sensitivity to others’ loss, which was in line with CLT and reflecting a modulation of social relationship in advice-giving outcome evaluation.

Some limitations and directions for further research need to be noted. Firstly, aligning with prior studies, advisors felt responsible for their advices, and became more guilty and regretful if the advisee accepted their wrong advices and thus received a loss (Leiser et al., 2008; Mobbs et al., 2015), while they might also experience negative emotion such as disappointment if advisee rejected their right advices and received a loss. Previous findings on the influence of emotions on decision-making have confirmed that subject’s choice preference was affected by emotional stimulus and emotional strategies under risk and uncertainty, and there was also an interpersonal effect of emotions in economic decision-making (Heilman et al., 2010; Wang et al., 2018). Our research only takes social relationship and outcome evaluation into account, and further examinations of the effect of subjective emotion experience on advice-giving may yield interesting findings. Secondly, the sample size in the present study was moderate overall, and we only recruited young college students for the experiment, which could limit the generalizability and replicability of our findings. In real life, business staff such as agents and brokers are more likely influenced by social relationships and feedbacks from their clients. Therefore, future studies are needed to examine our findings during the economic exchange. Thirdly, the present study adopted a pre-programmed computerized card-guessing task to simulate an advice-giving context. The advisor-advisee interaction was actually a cover story to subjects, although they all believed in the experimental settings before the formal experiment. However, advisors could not communicate with their partners and had no chance to exchange information, which differed from advisor-advisee interaction in real life. Prior studies have highlighted the influence of language expression and non-verbal communication during decision-making, and they found that face-to-face cues could reduce uncertainty and promote mutual intentionality, which would induce more prosocial behaviors (Tang et al., 2016; Jahng et al., 2017). To extend our findings, future research should explore the effect of social relationships on advice-giving in the social context with higher ecological validity, such as real-time interactive games, and advisors could use verbal and non-verbal cues to gauge interpersonal intentions. It would be useful to further understand the psychological mechanism of advice-giving.

## Conclusion

In summary, the present research is the first to investigate the modulation of social relationships on advisors’ social feedback processing and outcome evaluation in advice-giving. The FRN results showed enhanced neural sensitivity to social rejection from a stranger in the early stage of outcome evaluation. The P3 amplitudes were not only modulated by social relationships, but also affected by interpersonal social reward in the late stage. The friend-stranger difference on theta oscillation revealed differential sensitivity of theta power to other’s gain and loss, correlating with the balance of reward and social relationship. These findings contribute to our understanding of the neural mechanisms of advice-giving outcome evaluation in the context of social interaction.

## Data availability statement

The raw data supporting the conclusions of this article will be made available by the authors, without undue reservation.

## Ethics statement

The studies involving human participants were reviewed and approved by the Key Laboratory of Applied Brain and Cognitive Sciences at Shanghai International Studies University. The patients/participants provided their written informed consent to participate in this study.

## Author contributions

SX designed the experiment and provided the critical revisions. CZ, RT, HZ, KZ, and MD collected the data. RT and CZ analyzed the data with feedback from SX. CZ and HZ drafted the manuscript. All authors edited the manuscript.

## Footnotes

1
https://letswave.cn
